# Scaling DNA data storage with nanoscale electrode wells

**DOI:** 10.1126/sciadv.abi6714

**Published:** 2021-11-24

**Authors:** Bichlien H. Nguyen, Christopher N. Takahashi, Gagan Gupta, Jake A. Smith, Richard Rouse, Paul Berndt, Sergey Yekhanin, David P. Ward, Siena D. Ang, Patrick Garvan, Hsing-Yeh Parker, Rob Carlson, Douglas Carmean, Luis Ceze, Karin Strauss

**Affiliations:** 1 Microsoft Research, Redmond, WA, USA.; 2 Paul G. Allen School of Computer Science and Engineering, University of Washington, Seattle, WA, USA.

## Abstract

Synthetic DNA is an attractive medium for long-term data storage because of its density, ease of copying, sustainability, and longevity. Recent advances have focused on the development of new encoding algorithms, automation, preservation, and sequencing technologies. Despite progress in these areas, the most challenging hurdle in deployment of DNA data storage remains the write throughput, which limits data storage capacity. We have developed the first nanoscale DNA storage writer, which we expect to scale DNA write density to 25 × 10^6^ sequences per square centimeter, three orders of magnitude improvement over existing DNA synthesis arrays. We show confinement of DNA synthesis to an area under 1 square micrometer, parallelized over millions of nanoelectrode wells and then successfully write and decode a message in DNA. DNA synthesis on this scale will enable write throughputs to reach megabytes per second and is a key enabler to a practical DNA data storage system.

## Teaser

A demonstration of DNA synthesis control at over 1000x higher density shows the path to large scale DNA data storage.

## INTRODUCTION

**Fig. 1.**
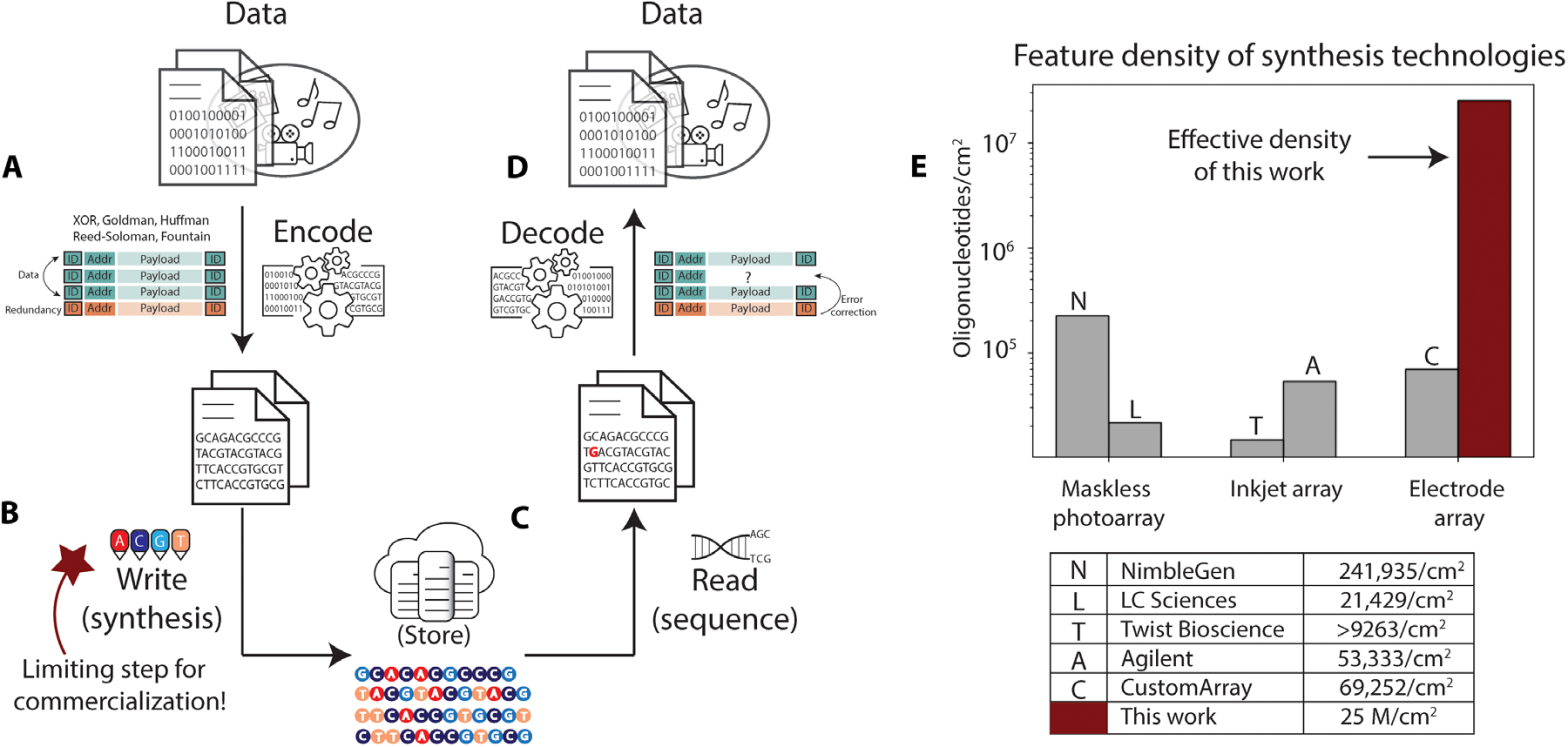
DNA data storage requires higher synthesis throughput than is possible with current techniques. (**A** to **D**) Overview of the DNA data storage pipeline. (A) Digital data are encoded from their binary representation into sequences of DNA bases, with an identifier that correlates them with a data object, addressing information that is used to reorder the data when reading, and redundant information that is used for error correction. (B) These sequences are synthesized into DNA oligonucleotides and stored. (C) At retrieval time, the DNA molecules are selected and copied via PCR or other methods and sequenced back into electronic representations of the bases in these sequences. (D) The decoding process takes this noisy and sometimes incomplete set of sequencing reads, corrects for errors and missing sequences, and decodes the information to recover the data. (**E**) Summary of the commercial synthesis processes and corresponding estimated oligonucleotide densities, as reported in the literature or by the companies themselves (see text S2). Our electrochemical method density is highlighted in dark red.

Data are being generated at a pace that exceeds current storage capacity. DNA is a promising solution to this storage problem because it is very dense, at an expected practical density of over 60 petabytes per cubic centimeter (*1*–*3*), very durable under the appropriate conditions (*4*–*6*), eternally relevant, easy to copy (*7*), and promises to be more sustainable than commercial media (*8*). These desirable properties have sparked substantial interest in the use of synthetic DNA as a digital data storage medium in large-scale deployments (*9*). Figure 1A illustrates the process of digital data storage using synthetic DNA. Digital data (i.e., sequences of bits) are typically encoded in sequences of the four natural DNA bases (*1*, *10*–*12*), although using additional bases is possible (*13*). These sequences are then “written” into molecular form through de novo DNA oligonucleotide synthesis, which creates the specified molecules via a set of repeating chemical steps using phosphoramidite chemistry (fig. S1). Once synthesized, the resulting oligonucleotides are preserved and stored. When data need to be accessed, the DNA storing it is selectively amplified using polymerase chain reaction (PCR) (*13*, *14*) or other methods (*15*, *16*) and sequenced, returning the DNA base sequences to the digital domain. Last, these DNA base sequences are decoded to recover the original sequence of bits.

A practical minimum throughput for writing digital data into DNA strands is in the kilobytes per second range, which is not achievable with existing synthesis infrastructure (*12*). Achieving the necessary parallel write throughput (not to be confused with latency or nucleotide incorporation time) while maintaining a realistic infrastructure footprint will thus require increasing the synthesis density, the number of different sequences that a single platform can synthesize per unit area. DNA synthesis parallelism is typically achieved through array synthesis techniques where each sequence is grown at a different site in the array simultaneously. The most space-efficient way to increase synthesis density is to reduce the area over which each unique sequence is grown (the feature size), the distance between features (the pitch), or both. Feature size limits the area required for generating one sequence, while pitch ultimately dictates the number of features at a given size that can be packed within an array. Smaller feature size and pitch result in higher synthesis density. High-density array synthesis amortizes the fixed costs of reagents and equipment over a larger number of oligonucleotides, which is reflected in the historic decrease in synthesis cost per base with the transition from column-based to array-based oligonucleotide synthesis (*17*).

Of existing DNA synthesis methods, photomask arrays have proven to generate the highest oligonucleotide densities according to the literature (*18*); however, this technique relies on a series of static, bespoke photolithographic masks to synthesize a defined set of sequences. Photolithographic masks at nanoscale features are costly, so this method is better suited to synthesizing repetitive DNA sequences than the arbitrary sequences required in a DNA storage system. The most successful approaches for the synthesis of arbitrary nucleotides yield substantially lower densities; Fig. 1E highlights the commercial densities achieved to date (*17*). Strategies using maskless digital photolithography or on-demand deposition (i.e., inkjet or acoustic synthesis techniques) enable rapid sequence customization, but the synthesis densities achievable with these techniques are limited by micromirror size and light scattering or droplet stability, respectively (*19*–*21*). Synthesis methods that use electrode arrays, on the other hand, leverage the scaling and production roadmap of the semiconductor industry, where features as small as 5 nm are now common. The instrumentation necessary for these strategies may be relatively simple, with the electrode array chip serving as both controller and substrate for DNA synthesis.

## RESULTS

We have produced an electrode array and demonstrated independent electrode-specific control of DNA synthesis with electrode sizes and pitches that enable synthesis density of 25 million oligonucleotides/cm^2^, the estimated electrode density required to achieve the minimum target of kilobytes per second of data storage in DNA (see text S2) (*12*). Our work pushes the state of the art in electronic-chemical control, outpaces the previously reported densest synthesis of arbitrary DNA sequences by a margin of three orders of magnitude, and provides the first experimental indication that the write bandwidth required for DNA data storage can be achieved.

**Fig. 2.**
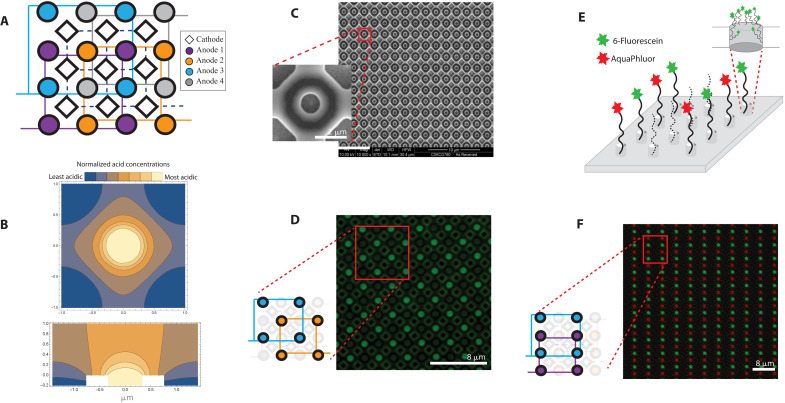
Overview of 650-nm array pitched 2 μm. (**A**) Finite element analysis of anodic acid generation and diffusion at a 650-nm-diameter electrode with a 200-nm well is depicted with a cross-sectional view along the *y* = *x* plane and (**B**) top-down view on the *z* = 0 plane. The colors blue and yellow represent regions with relatively low and high acid concentrations, respectively. (**C**) An overview of the nanoscale DNA synthesis array with scanning electron microscopy images of the 650-nm electrode array and enlarged view of one electrode. (**D**) A fluorescent image in which the well surrounding each activated anode is patterned with AAA-fluorescein. The cartoon diagram depicts which electrodes in the layout were activated. (**E**) Illustration of the wells patterned with AAA-fluorescein and AAA-AquaPhluor and (**F**) corresponding image overlay of the two fluorophores at the end of DNA synthesized on the same 650-nm electrode array.

In electrochemical phosphoramidite synthesis, the electrode activation state during the deblock step controls whether a new base will be added in the next coupling step: An activated electrode forms acid locally and removes the protecting groups attached to the oligonucleotides growing at that site, freeing it for the next coupling. A major concern when scaling down the electrode pitch in this process is acid diffusion (*22*, *23*). The smaller the pitch, the closer the electrodes are, and the easier it is for acid generated at one electrode to diffuse to neighboring electrodes. This may cause unintended deblocking of DNA at neighboring electrodes, resulting in insertion errors in the final sequence. While we had success in preliminary experiments containing acid to the generating electrodes at scales greater than a micrometer (fig. S11), it was not clear that the trend would hold to indefinitely small feature sizes. Thus, when designing the electrode arrays used in this study, we adopted a layout where each working electrode, the anode where the acid formation happens in phosphoramidite synthesis, is sunk in a well and surrounded by four common counter electrodes, cathodes that drive base formation, to confine the acid to the region immediately around the anodes. This was intended to provide both physical and chemical barriers to control acid diffusion (*23*). We verified the effectiveness of this design in confining acid by modeling the acid generation and diffusion of a 650-nm anode pitched 2 μm at steady state (see text S3). Our finite element analysis indicated that acid is present inside the anode region at a much higher concentration than the cathode region (Fig. 2, A and B), suggesting that our design is effective at acid confinement.

The electrode dimensions of the array (Fig. 2C) were dictated by the smallest achievable feature size that the technology process node could provide. To verify acid confinement to the generating electrode experimentally, we had an array of 650-nm electrodes manufactured (see text S4) and performed a fluorescence assay using fluorophore-labeled phosphoramidites. First, we covered the entire surface of the array with a 5′-trityl–protected nucleotide. Next, we activated anodes at +1.8 V relative to the cathodes for 60 s in a checkerboard pattern, as shown in Fig. 2D cartoon, to generate acidic microenvironments around the activated anodes. When present in sufficient concentration, the acid deblocks the surface-bound nucleotides and enables the next nucleotide to couple. Last, the deblocked strands were extended with another 5′-trityl–protected nucleotide. We extended the growing strands four times, with three additions of adenosine followed by one of a terminal fluorophore-labeled phosphoramidite, to achieve the pattern observed in Fig. 2D. Strict confinement of fluorescence to the activated electrodes indicated that the electrochemically generated acid was similarly confined. To demonstrate independent control of the anodes, we parallelized the synthesis process to generate two different sequences as envisioned in Fig. 2E: AAA-fluorescein for one anode (green) and AAA-AquaPhluor for a second (red). The resulting array showed the two fluorophores confined to their respective electrodes (Fig. 2F).

Having demonstrated spatially controlled synthesis of short oligonucleotides on our electrode array, we next sought to evaluate the maximum length of DNA that could be synthesized. To this end, we created a single DNA sequence that was 180 nucleotides (nt) long as a concatenation of nine distinct 20-nt sequences designed to be used as primer attachment sites (see text S6). The predicted annealing temperatures of these nine primer sequences were tightly clustered, allowing us to use internal sites to amplify sections of the composite 180-length oligonucleotide in 20-nt intervals. We synthesized the full 180-length oligonucleotide using electrochemically generated acid deblocking on the 650-nm array, with all four anodes producing the same sequence. After cleavage from the array, we PCR-amplified the oligonucleotide using the 3′ reverse primer and appropriate forward primers to yield oligonucleotides ranging from 60 to 180 nucleotides in length (fig. S9), indicating a full-length product with the correct sequence. We then sequenced the PCR products to assess quality.

**Fig. 3.**
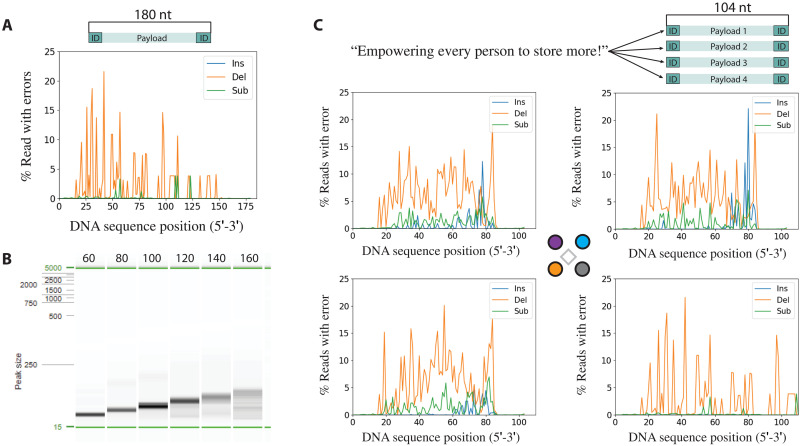
Errors stemming from synthesis followed by sequencing. (**A**) Insertions (Ins), deletions (Del), and substitutions (Sub) per position for a synthesized and PCR-amplified 180-base sequence. (**B**) Electrophoresis image of synthesis products after PCR amplification. (**C**) Message encoded into 64 bytes split into four unique sequences of 104 bases (top). Insertions, deletions, and substitutions per locus of each of the four sequences in the multiplex synthesis run. In every error analysis graph, the terminal 20 bases at both 3′ and 5′ ends come from the primers used in PCR and are not representative of the synthesized errors.

Figure 3A shows gel electrophoresis of the various-length PCR products amplified from the complete 180 oligonucleotides. As the amplicon gets longer, the expected PCR product appears fainter and less well defined, while the amplicons at lengths of 100 or fewer show stronger and more well-defined bands, which is indicative of higher synthesis errors at the 5′ end of the oligonucleotide. This is likely due to an increase in electrochemical cell resistance added by steric hindrance from longer DNA (*24*), which grows from 3′ end to 5′ end. While the full-length 180 amplicons was difficult to amplify and appeared very faint, we were able to sequence it to confirm our hypothesis. Error rates in Fig. 3B are computed on the basis of all reads that aligned to our intended 180-base reference sequence. Figure 3B shows that the dominant type of error is deletions, with deletion rates rising toward the 5′ end of the sequence, which likely affected our ability to amplify the longer sequences cleanly. The increased error rate is attributable to an increase in cell resistance with the presence of increasingly longer DNA immediately around the electrodes, grown from 3′ end to 5′ end (fig. S10). An average of 3.2 deletions were observed per read (table S1), corresponding to a deletion rate of 1.8% across the full reference (2.2% excluding the primer regions). Substitutions and insertions were much less common and do not create data recovery concerns (*12*). On the basis of these results, we selected a sequence length of around 100 bases for ease of purification with which to provide a practical demonstration of DNA data storage and continued without any further optimization.

Last, we demonstrate that the quality of DNA synthesized on our array was sufficient for DNA data storage. A 40-byte message (“Empowering every person to store more!”) was encoded into four unique 64-nt payloads sharing a common primer pair, and the resulting 104-nt sequences were synthesized in parallel on a single 650-nm-diameter electrode array. Each of the four sequences was assigned to an anode, and at each synthesis cycle, we selectively activated subsets of the four anodes, which allowed us to independently control nucleotide additions at each site according to the unique sequence to be constructed there.

Following cleavage from the surface, the oligonucleotides were amplified and sequenced. Figure 3C shows the error rates over all cycles for each of the four DNA sequences we synthesized. These sequences experience higher error rates than the single sequence we synthesized in the previous experiment (Fig. 3B). Again, deletions were the most prevalent errors at an average rate of 4.3% per full-length read (6.3% excluding the primer regions), increased from the rates observed with a single sequence synthesis. Insertion and substitution rates are also more common, at 0.45% and 0.94%, respectively. This is likely caused by inadequate washing of the array between synthesis steps, leading to exposure of the DNA sequences to bases not intended to be added during off-cycles as opposed to electrode-to-electrode cross-talk, given that previous experiments showed confinement of the deblock reaction to the initiating electrode (Fig. 2, D and F). Nonetheless, despite the higher errors, we were able to sequence and decode the 40-byte message exactly as originally encoded, with no bit errors.

## DISCUSSION

By using an electrochemical array, we have achieved the first parallel synthesis of arbitrary DNA sequences at nanoscale feature sizes that can yield densities high enough to be useful for DNA data storage. Relative to existing electrochemical array–based DNA synthesis, we have achieved a three–order-of-magnitude increase in density, tripled the length of oligo synthesized (*21*, *22*), and maintained error rates compatible with DNA data storage. The synthesized oligos contained deletion, substitution, and insertion errors at a cumulative rate of 4 to 8% per synthesized base (Fig. 3C).

The observed error rates are well within the acceptable range for modern error correcting codes for data storage in DNA (*25*, *26*), where average error rates as high as 15% have been shown to be tolerable (*27*, *28*). Furthermore, using the combination of physical redundancy and clustering algorithms to generate consensus sequences will yield effective error rates lower than the reported per-base error rate. We expect additional gains in per-base error rates to come with optimizations to the composition of the deblocking solution, the deblocking and addition cycle times, and the fluid delivery platform.

While the electrode densities used in these experiments were limited by the 130-nm process node used to produce the microelectrode array, we project that the technology will scale further to billions of features per square centimeter, enabling synthesis throughput to reach megabytes-per-second levels in a single write module, competitive with the write throughput of other storage devices. As an additional benefit, since synthesis operations within each module happen in parallel, increases in the synthesis density amortize the cost of reagents across more reaction sites and substantially reduce the cost per DNA sequence. Last, in addition to their immediate application to information technology, nanoscale electrochemical synthesis arrays enable high-throughput conversion of digital signals into molecular structures, opening up the field of electronically controlled, high-throughput matter assemblers. We foresee these assemblers being used in other areas like material science, synthetic biology, diagnostics, and closed-loop massive molecular biology experimental assays.

## MATERIALS AND METHODS

### Synthesis and sequencing

Fluid delivery was handled using an Expedite 8900 oligonucleotide synthesizer. All reagents, with the exception of the deblock, were standard for phosphoramidite chemistry and used as recommended by the manufacturer. The alternative deblock solution was composed of 50 mM hydroquinone, 50 mM tetraethylammonium *p*-toluenesulfonate, and 2.5 mM anthraquinone in a 9:1 mixture of acetonitrile and methanol.

Coupling, capping, and oxidation synthesis cycles were performed using the default fluidics protocols for column synthesis on 50-nmol scale. Deblocking cycles were performed using a modified protocol, which incorporated a triggering pulse to sync exposure to electrochemical deblock solution with application of voltage (fig. S3). Fluids were supplied to the nanoelectrode array surface via a two-piece, stainless steel flow cell. Polyether ether ketone (PEEK) polymer fittings allowed access to an approximately 100-μl cavity bounded by an ethylene propylene diene monomer (EPDM) polymer gasket. Electronic control of the microelectrode array is achieved via peripheral component interconnect express (PCIe) connectors exposed outside of the flow cell (fig. S3).

Once the synthesis protocol was complete, DNA was cleaved off the surface of the chip using 32% ammonium hydroxide and deprotected overnight at 65°C. The solution was then concentrated to dryness in a SpeedVac vacuum concentrator, followed by resuspension in 40 μl of molecular biology–grade water. The DNA was amplified using PCR and purified using a Qiagen QIAquick spin column or gel extracted as needed. The enriched DNA was then amplified for a second time with primers containing random 25-N overhangs, ligated, and sequenced using an Illumina NextSeq.

Sequences were aligned using a modified Bitwise Majority Alignment algorithm (*29*). More details regarding usage of the 25-N overhangs, ligation protocol, sequencing preparation, and error analysis are described in the work of Organick *et al.* (*12*).

### Encoding

The American Standard Code for Information Interchange (ASCII) bytes representing the string Empowering every person to store more! were split into eight substrings, 5 bytes each (40 bits), and encoded into 32-base DNA sequences using the same method as in our previous work (*30*). Consecutive pairs of DNA sequences were concatenated into 64-base sequences, and common 20-base 5′ and 3′ primer sequences were added to each, creating four 104-base strands to be synthesized.

### Decoding

Raw sequencing data were clustered and trace-reconstructed as in our previous work (*12*), and full-length DNA sequences corresponding to the four largest clusters were recovered. Next, for each of the DNA sequences, we verified whether it formed a valid codeword according to the encoding algorithm (*30*). For DNA sequences that did not represent valid codewords, we exhaustively enumerated all sequences within edit distance two or less (i.e., all sequences that can be obtained by one or two substitutions or by a combination of an insertion and a deletion). In cases when the resulting set of sequences contained a unique valid codeword, we applied the appropriate error correction to obtain the original DNA sequence. This process allowed us to successfully recover all four encoded sequences.

## Supplementary Materials

### This PDF file includes:

Supplementary Text Figs. S1 to S11 Table S1 References

## Notes

View/request a protocol for this paper from *Bio-protocol*.
