# Supplementary material for: Scaling DNA data storage with nanoscale electrode wells
Source: Sci Adv. 2021 Nov 24;7(48):eabi6714. doi: 10.1126/sciadv.abi6714 (PMC8612674; doi:10.1126/sciadv.abi6714)
Supplement: Supplementary file 1 — Supplementary Text Figs. S1 to S11 Table S1 References [file sciadv.abi6714_sm.pdf]

Science Advances
AAAS

Supplementary Materials for

Scaling DNA data storage with nanoscale electrode wells

Bichlien H. Nguyen*, Christopher N. Takahashi, Gagan Gupta, Jake A. Smith, Richard Rouse, Paul Berndt, Sergey Yekhanin, David P. Ward, Siena D. Ang, Patrick Garvan, Hsing-Yeh Parker, Rob Carlson, Douglas Carmean, Luis Ceze, Karin Strauss*

*Corresponding author. Email: bnguy@microsoft.com (B.H.N.); kstrauss@microsoft.com (K.S.)

Published 24 November 2021, *Sci. Adv.* **7**, eabi6714 (2021)
DOI: 10.1126/sciadv.abi6714

This PDF file includes:

Supplementary Text
Figs. S1 to S11
Table S1
References

S1 Phosphoramidite Synthesis Setup

Materials: Standard reagents (activator, cap A and B, oxidizer, anhydrous acetonitrile, and 32% ammonium hydroxide), 6-fluorescein phosphoramidite, hydroquinone, anthraquinone, tetraethylammonium p-toluene sulfonate, and methanol were purchased from Sigma-Aldrich. Standard phosporamidites, acetonitrile diluent, and AquaPhluor 593 phosphoramidite were purchased from Glen Research. Universal linker phosphoramidite was purchased from AM Chemicals as 100mg prepackaged vials. Consumables (trap packs, o-rings, bottles) were purchased from BioAutomation.

Mechanisms:

Generalized phosphoramidite synthesis

Phosphoramidite synthesis occurs via repeated four-step cycles (Figure S1 (a)). In the first cycle (deblocking), the 2,2'-dimethoxytrityl protecting group is removed from the 5' end of the growing oligonucleotide strand by exposure to acid. In the second (coupling), an additional nucleotide phosphoramidite is added to the newly free 5' end. In the third (capping), free 5' ends of non-reacted strands are "capped" with acetic anhydride to prevent further extension. This capping step may be excluded in the case of very short oligonucleotides or with sufficiently high coupling efficiencies. In the fourth and final step (oxidation), the newly formed phosphite backbone linkage is oxidized to the phosphate, improving its stability to future cycles of synthesis. Additional repetitions of this four-step cycle result in synthesis of oligonucleotides of arbitrary sequence.

Electrochemical generation of acid

During the electrochemical deprotection process, acid was generated from a two-electron oxidation of hydroquinone to quinone at each activated anode, and methoxide anion was generated from reduction of methanol at each cathode (Figure S1(b)) (*22, 34*). The electrochemically generated acid was free to diffuse until it reached a basic methoxide anion in solution and was neutralized, effectively confining the acid to the region immediately around the generating electrode. Only where sufficient acid was present would significant deblocking of oligonucleotides occur.

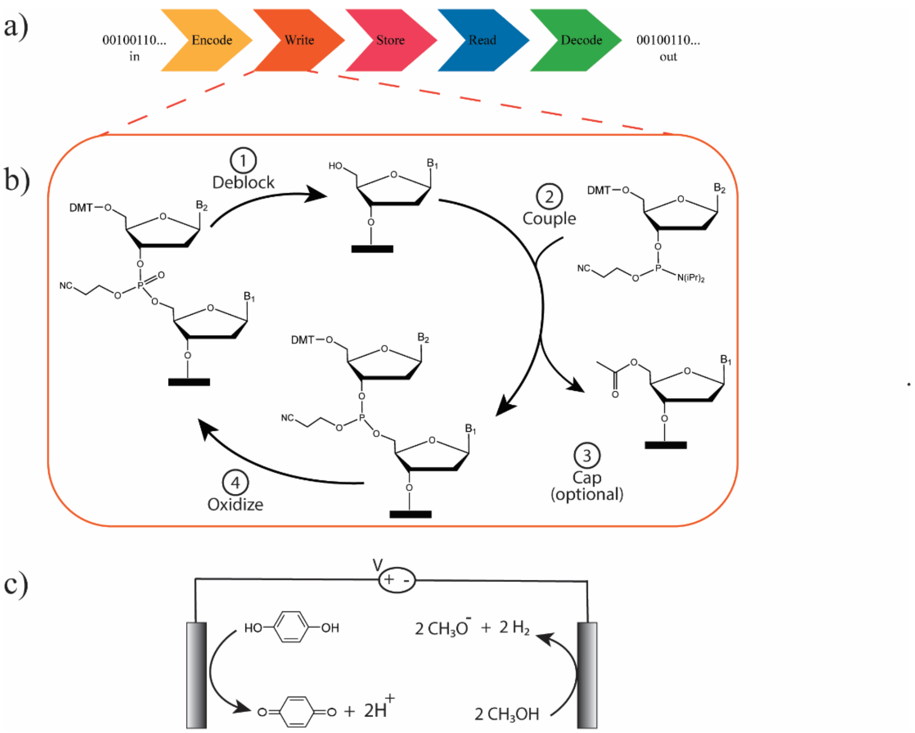

Fig. S1: **Writing data in DNA with electrochemically mediated phosphoramidite synthesis.** a) The DNA data storage pipeline. b) Phosphoramidite synthesis steps and reactions. The four-step cycle used in phosphoramidite synthesis. c) Half-reactions occurring at the anode and cathode during the electrochemical deblock step.

Function		Mode	Amount /Arg1	Time(sec) /Arg2	Description
$Deblocking					
144	/Index Fract. Coll	/NA	1	0	"Wait"
0	/Default	/Wait	0	1.5	"START data collection"
141	/Trityl Mon. On/Off	/NA	1	1	"Aux"
17	/Aux	/Pulse	45	0	"Event out ON"
0	/Default	/Wait	0	60	"Wait for electro"
38	/Diverted Wsh A.	/Pulse	35	60	
141	/Trityl Mon. On/Off	/NA	0	0	
144	/Index Fract. Coll.	/NA	2	1	

Fig. S2: **A representative Expedite protocol for electrochemical deblocking.** Coupling, capping, and oxidation synthesis cycles were performed using the default fluidics protocols for column synthesis on 50 nmol scale. Deblocking cycles were performed using a modified protocol which incorporated a triggering pulse to sync exposure to electrochemical deblock solution with application of voltage (Figure S2). Fluids were supplied to the nanoelectrode array surface via a two-piece, stainless steel flowcell. PEEK fittings allowed access to an approximately 100 µL cavity bounded by an EPDM gasket. Electronic control of the microelectrode array is achieved via PCIe connectors exposed outside of the flow cell (Figure S3).

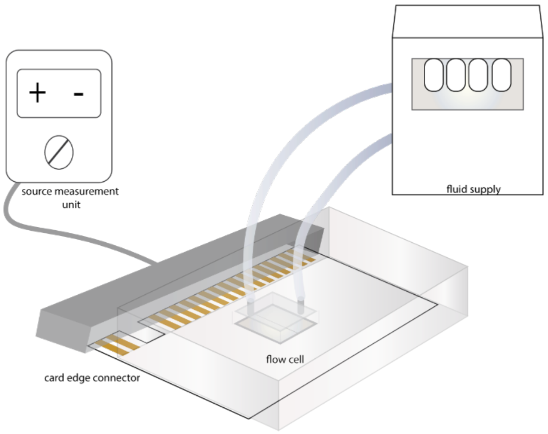

Fig. S3: **Two-piece flow cell design.**

S2 DNA data storage throughput calculations

To achieve write speeds of kB/s of data in DNA, an array containing >8.8 million spots for DNA synthesis is required assuming each unique oligonucleotide encodes for 10 bytes of data [1] and is written over 24 hours. With these assumptions, write speeds of MB/s of data in DNA would require >8.8 billion unique oligos written every 24 hours. In order to minimize the array footprint as the required number of features increases to match target outputs, the pitch between the features becomes critical. An array with a 2µm pitch can fit 25 million features per cm^2, write >2.8 $kB/s/cm^2$, and would require roughly 360 cm^2 to write approximately 1 MB/s. Feature densities of the commercial DNA synthesis platforms were estimated by numbers provided by the company directly from their respective website or online presentations (*31-33*).

NimbleGen: 2.1M array, 62mm x 14mm
LC Sciences: 30k array, 1.4 cm^2
Twist: >1M array, 127mm x 85mm
Agilent: 1M array, 75mm x 26mm
CustomArray: 40µm pitch

S3 Acid generation and diffusion modeling

We performed a basic finite element analysis of a 650nm diameter electrode pitched 2µm to test for sufficient acid confinement. We modeled a single electrode tile area with zero flux boundary conditions along the non-conducting areas as well as the tessellation boundaries. Electrode surfaces were set to values proportional to their relative area to account for the balanced generation rates on the anodes and cathodes. Since values for the acid/base species we used in this work were not available in the literature, we chose a value close to the middle of the range for other small molecules: 9×10^{-5} cm^2/s.

Science
AAAS

Definition of boundary value problem

$$conc_{H+} = u(t,x,y,z) \qquad \frac{\partial u}{\partial t} = 0 = D_u \nabla^2 u$$

$$u_t(t, \tfrac{-L_x}{2}, y, z) = 0 \qquad u_t(t, x, \tfrac{-L_y}{2}, z) = 0$$

$$u_t(t, \tfrac{L_x}{2}, y, x) = 0 \qquad u_t(t, x, \tfrac{L_y}{2}, z) = 0 \qquad u_t(t, x, y, L_z) = 0$$

$$\begin{cases} u_t(t,x,y,-0.2) = 4, & \text{for } x, y \text{ over anode} \\ u_t(t,x,y,-0.2) = 0, & \text{for } x, y \text{ not over anode or cathode} \\ u(t,x,y,-0.2) = 0, & \text{for } x, y \text{ over cathode} \\ u_t(t,x,y,z) = 0, & \text{for } x, y, z \text{ on well walls} \end{cases}$$

Mathematica code for simulation

```
*import finite elements solver*
Needs["NDSolve`FEM`"]

*draw a unit cell of the electrode array from geometric shapes*
(*a=ImplicitRegion[True,{{x,-1,1},{y,-1,1},{z,0,1}}];*)

a = Cuboid[{-1, -1, 0}, {1, 1, 1}];
b = Cylinder[{{0, 0, -1/5}, {0, 0, 0}}, (650/1000)/2];
c = Cylinder[{{1, 1, -1/5}, {1, 1, 0}}, 650/1000];
d = Cylinder[{{-1, 1, -1/5}, {-1, 1, 0}}, 650/1000];
e = Cylinder[{{1, -1, -1/5}, {1, -1, 0}}, 650/1000];
f = Cylinder[{{-1, -1, -1/5}, {-1, -1, 0}}, 650/1000];
r = RegionUnion[a, b, c, d, e, f];
(*boundingbox=ImplicitRegion[True,{{x,-1,1},{y,-1,1},{z,-1/5,1}}];*)

boundingbox = Cuboid[{-1, -1, -1}, {1, 1, 1}];
r2 = RegionIntersection[r, boundingbox];

*construct a mesh from the unit cell*
mesh = ToElementMesh[r2, "BoundaryMeshGenerator" -> {"OpenCascade"}];

*visualize the mesh*
groups = mesh["BoundaryElementMarkerUnion"];
temp = Most[Range[0, 1, 1/(Length[groups])]];
colors = ColorData["BrightBands"][#] & /@ temp;
mesh["Wireframe"["MeshElementStyle" -> FaceForm /@ colors]]

*set flux through the anode to a constant value*
nv = NeumannValue[4, (x)^2 + (y)^2 < 1.01 (650/1000/2)^2 && z == -1/5];

*set acid concentration at the cathode surface to zero*
dc = DirichletCondition[u[x, y, z] == 0, (x)^2 + (y)^2 > 1.01 (650/1000/2)^2 && z
    == -1/5];

*boundary conditions not explicity stated default to zero flux*

*write out the PDE*
*diffusion coefficient approximated at 9000 um/s*
op = Inactive[Div][{{-9000, 0, 0}, {0, -9000, 0}, {0, 0, -9000}}.Inactive[Grad][
  u[x, y, z], {x, y, z}], {x, y, z}];

*solve the IVBP over the mesh*
ufun3d = NDSolveValue[{op == nv, dc}, u, {x, y, z} \[Element] mesh];

*plot the steady-state solution "top-down"*
ContourPlot[ufun3d[x, y, 0] - m, {x, -1, 1}, {y, -1, 1},
  ClippingStyle -> Automatic, AspectRatio -> Automatic,
  PlotLegends -> Automatic, PlotPoints -> {75, 75}]
  Export["top_plot.pdf", %]

*plot the steady-sate solution "side-on"*
ContourPlot[
  ufun3d[xy/Sqrt[2], xy/Sqrt[2], z] - m, {xy, -Sqrt[2],
  Sqrt[2]}, {z, -0.2, 1}, ClippingStyle -> Automatic,
  AspectRatio -> Automatic, PlotLegends -> Automatic,
  PlotPoints -> {75, 50}]
```

Science
AAAS

S4 Chip Fabrication and Electrode Activation

A silicon wafer containing an array of 650nm diameter electrodes pitched 2µm was manufactured using standard nanolithography 130nm process technology. The wafer was diced and mounted on a FR4 PCB. The A and B face of the PCB were mirrored and designed for a high-density card interface. The die pins were wire bonded to their corresponding traces on the PCB and then protected by an epoxy encapsulate to create the slide assembly. A flow cell assembly was designed in two pieces. The bottom piece was fitted with two locating pins to sandwich the chip assembly in place with the top piece, which contained a recessed gasket. The top piece contained 2 I/O ports to connect inline with an Expedite 8900 oligonucleotide synthesizer. To address the electrodes on the array, a card edge connector was connected to the slide assembly driven by a National Instruments PXIe-4141 Source Measure Unit.

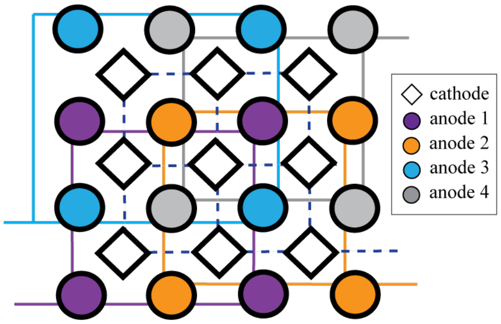

Fig. S4: **Illustration of the electrode array schematic.** Circles represent the anodes; diamonds represent the cathodes. Cathodes are connected together (represented by the dashed line) while only anodes of the same color are connected together to generate four addressable electrodes (represented by the similarly-colored jumpers – solid lines)

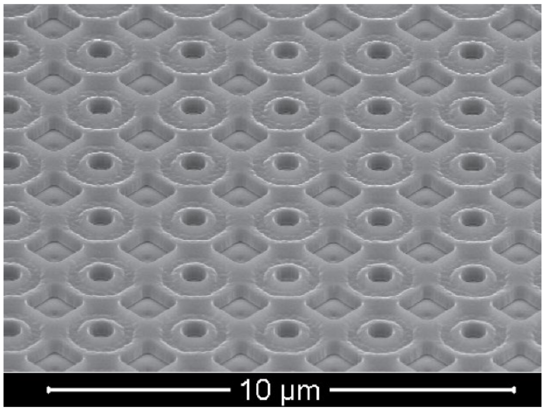

Fig. S5: **Tilted SEM image view of the electrode array.** The circles are the anodes and diamonds are the common cathodes.

S5 Imaging

SEM images were taken with a FEI Quanta 600 SEM coupled with an EDS detector. Fluorescent images were taken on either an Olympus BX53MTRF-S equipped with a DP74 color CMOS camera or Leica DM8 Lightning confocal system. Image processing was performed using ImageJ. Image analysis of fluorescence on 650 nm electrodes showed a toroidal profile which indicates that oligonucleotide synthesis occurs on the walls of the SiO_2 well, leaving the electrode surface free of fluorescence.

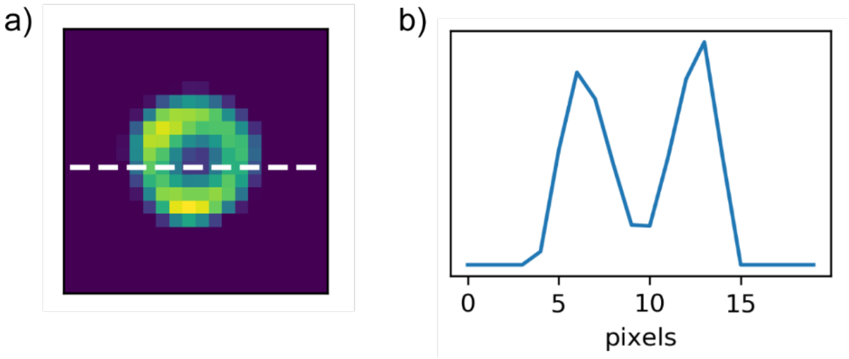

Fig. S6: **Fluorescence image of sequence AAA-[fluorescein].** Synthesized on a single 650nm electrode acquired on a Leica SP8 microscope with a 25x, 0.95 NA water immersion objective (a) and corresponding fluorescence intensity profile (b).

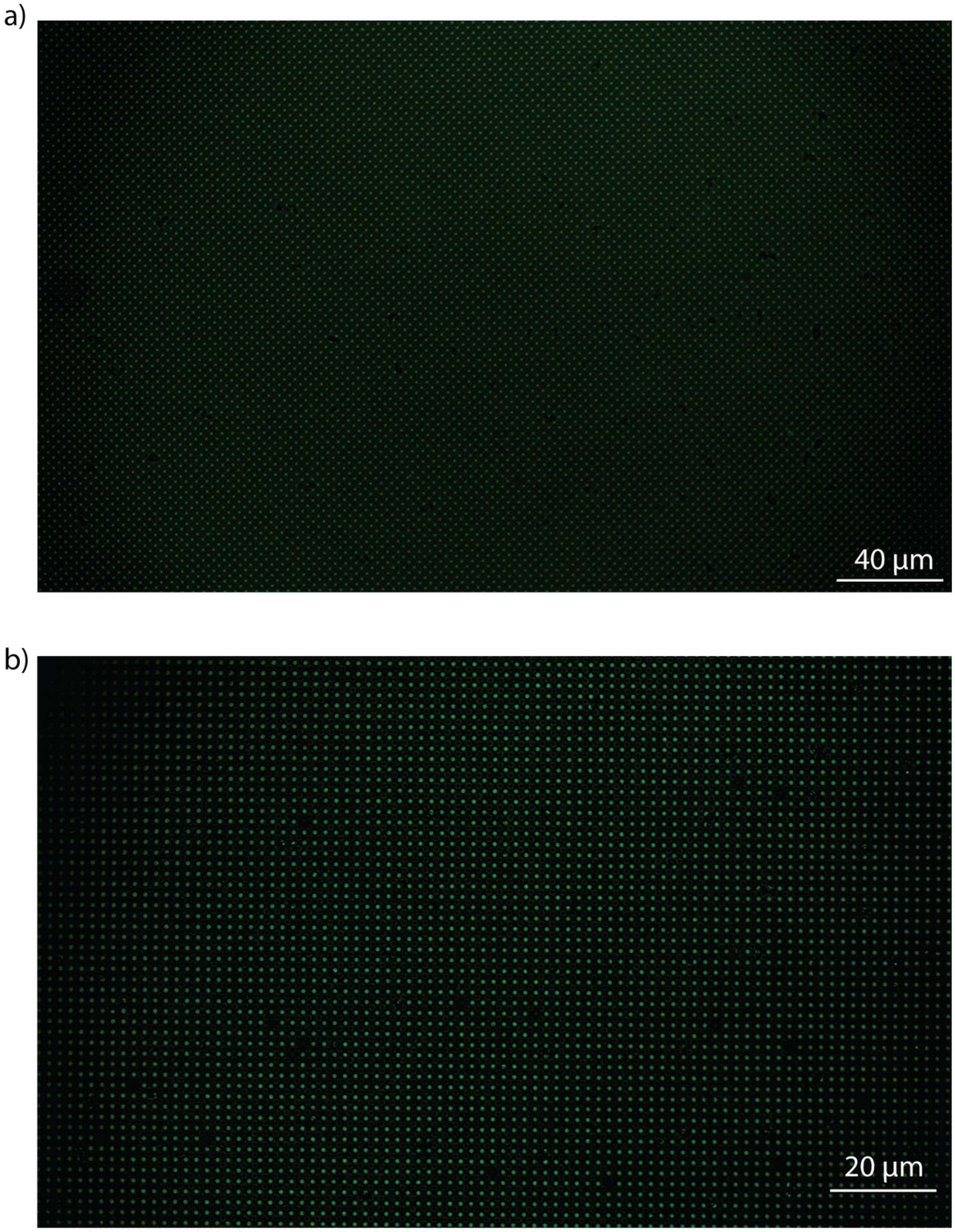

Fig. S7: **Fluorescence image of sequence AAA-[fluorescein].** Synthesized on a 650 nm electrode array acquired on an Olympus BX53MTRF-S demonstrating fluorescence uniformity across large section of the electrode array. a) Pattern was generated with 1 anode activated and imaged using a 50x objective b) Pattern generated with all 4 anodes activated and imaged using a 100x objective. Anode locations that are dark indicated an electrode fabrication failure.

S6 Array Synthesis Experiments

Master Sequence Generation: The master sequence and electrode activation sequencing are constructed by first only considering the payload sections (i.e., the sequences without the common primer pair on the 5' and 3' ends.). A periodic master sequence of sufficient length is then generated by repeating the symbols CAGT (or a shuffled version of the same characters). Electrode sequencing was determined for each electrode based on the next base is the 3'-5' sequence to be generated. At cycle N, if the Nth character in the master sequence is the same as the 3' most unincorporated base for the sequence associated with electrode a particular electrode, then that electrode is activated causing a base to be incorporated. Next the 20-base primer sequences are added to the 3' and 5' end of the master sequence as well as 20 3' and 5' electrode activations to ensure the primer sequence is incorporated. Finally, any cycles with 0 activations are removed.

This method ensures that no more than $4N + 20 \times 2$ cycles are required for any set of sequences with payload length N and primer length 20. While this algorithm is not optimal, we found it to be sufficient and that as the number of unique sequences increases the number of cycles needed approaches the upper limit above. We conjecture that finding the optimal master sequence is equivalent to finding the global multisequence alignment of all sequences to be synthesized with infinite mismatch penalty and gap penalty >0.

Science
AAAS

Python code for master sequence generation.

```python
import numpy as np
import json

base_cycle = list("CAGT")
FP = "CCGCATCAGATCGTATCCAT"
RP = "GGACTGAAACGATGGCTTGA8"
electrodes = np.array(["1S","2S","3S","4S"])

inputsequences = "seq.txt"

def is_empty_lol(lst):
  """ checks for empty list of lists"""
  for L in lst:
    if len(L)>0:
      return False
  return True

def parse_sequences(fname, fp=None,rp=None):
  ### Load sequences
  sequences = []
  with open(fname,"rb") as inf:
    for line in inf:
      _sq = list(line.strip().upper())
      if len(_sq)>0:
        sequences.append( _sq )
  #make sure we have one sequence for each electrode used
  assert len(sequences)==len(electrodes)

  ###Generate Master
  master_index = 0
  master = []
  echem = [[] for _ in range(len(sequences))]

  #while there are still some bases to process.
  while not is_empty_lol(sequences):
    current_base = base_cycle[master_index%len(base_cycle)]
    master_index +=1
    master.append(current_base)
    #for every electrode/sequence pair:
    for (_e,_s) in zip(echem, sequences):
      #if the 3' most base of the sequence == the current cycele's base
      #then add a 1 to turn the electrode on this cycle, otherwise add a 0
      if len(_s)>0 and (_s[-1] == current_base):
        _e.append(1)
        _s.pop()
      else:
        _e.append(0)

  ###add primers
  master = list(fp.upper())+master[::-1]+list(rp.upper()) #this is now
   5'-3'
  echemwp = [ [1]*len(rp)+x+[1]*len(fp) for x in echem ]
  echem_c_s = np.transpose(np.array(echemwp)) #[cycle][sequence]

  #distribute between electrodes
  cycle_actions = zip(*echemwp)
  #do some basic optimization
  opt_master = []
  opt_cycles = []
  master.reverse()#3'-5'
  for (a,s) in zip(cycle_actions,master):
    if sum(a) >0:
      opt_master.append(s)
      opt_cycles.append(a)
  master = opt_master
  master.reverse()#5'-3'
  cycles = []
  for cycle in opt_cycles:
    c = np.array(cycle,dtype=np.bool)
    cycles.append(electrodes[c])
  print master
  #for ii in cycles:
  #  print ii
  cycles = map(list,cycles)
  print json.dumps(cycles,indent=2)

  with open("master_seq.txt","wb") as outf:
    outf.write("".join(master))
  print len(master)
  print len(cycles)

if __name__ == '__main__':
  parse_sequences(inputsequences,FP,RP)
```

Error analysis: A high-level description of the end-to-end math pipeline used in Fig. 3

[Step 1] Perform an alignment on the sequencing reads. This generates an alignment file in SAM format containing a CIGAR string for each aligned read. Here's an example CIGAR string: "25S18M1D26M3I27M79S".

This CIGAR string can be read like this:
+ 25S = SKIP: skip over the first 25 bases in the read
+ 18M = MATCH: the next 19 bases in the read match the bases in the reference. n.b. A "Match" can be a sequence match **or** a sequence mismatch; aka a substitution.
+ 1D = DELETION: the next 1 base in the reference is missing from the read
+ 26M = MATCH: the next 26 bases in the read match bases in the reference.
+ 3I = INSERTION: the next 3 bases in the read are not present in the reference
+ 27M = MATCH: the next 27 bases in the read match bases in the reference
+ 79S = SKIP: skip over the next 29 bases in the read

[Step 2] Read in the alignment file containing the CIGAR strings and accumulate the CIGAR strings for each reference into three arrays.

For each reference, create:
+ One array capturing the total INS errors for each position in the reference oligo
+ One array capturing the total DEL errors for each position in the reference oligo
+ One array capturing the total SUB errors for each position in the reference oligo

For each reference, this step also accumulates the total count of reads which aligned with the reference. i.e. The total count of CIGAR strings for the reference.

[Step 3] The INS, DEL, and SUBS error arrays for all references are then combined into a single set of three arrays:
+ Total INS errors for each oligo position for all references
+ Total DEL errors for each oligo position for all references
+ Total SUB errors for each oligo position for all references

This step also accumulates the total count of aligned reads across all references. i.e. The total count of CIGAR strings across all references.

[Step 4] The elements in the Total INS, Total DEL, and Total SUB arrays are divided through by the total read count, and multiplied by 100 to give:
+ An array containing the % reads with INS error
+ An array containing the % reads with DEL error
+ An array containing the % reads with SUB error

These three arrays are charted together.

	# of reads	Average INS / Read	Average DEL / Read	Average SUB / Read
180 mer	352608	0.0008	3.1664	0.2640
180 mer (payload only)		0.0003	3.1227	0.2476
104 mer	754721	0.4687	4.4297	0.9734
104 mer (payloads only)		0.4565	4.0179	0.9280

Table S1: Average INS, DEL, and SUBS per read are listed for the 180 mer and 104 mer synthesized on the array.

Correlation of errors in multisequence synthesis: There is a statistically significant association between error rates and base locations, excluding the primer regions, X^2 (141, N = 25832266) = 444046, $p < 2.2e{-16}$; however, the effect size is minimal, Cramer's $V = 0.0757$. The minimal effect size indicates that the error rates were likely the result of independent random processes, given the tendency of the X^2 test to overestimate significance at high sample numbers. No statistically significant correlation was found between cumulative error rates of sequences at positions in which electrodes were activated during the same synthesis cycle, with $r = 0.093$ and $p = 0.125$.

Sequences: A, C, T, and G represent dA-CE, dC-CE, dT-CE, and dG-CE phosphoramidites, respectively. 6 represents 6-fluorescein phosphoramidite. 5 represents AquaPhluor 593 phosphoramidite. 8 represents universal linker phosphoramidite.

Single fluorophore experiment
5'-6AAA-3'

Dual fluorophore experiment
5'-5AAA-3'
5'-6AAA-3'

Maximum length synthesis experiment
5' -^{1}TCTTCGGATCACGGTCTTTG ^{21}ATTAGCGCAACTTCCACGAA
^{41}ACAAACTCATGGCTCCGTTT ^{61}CCAGAATCGTGCCTTCCTGG
^{81}GTCCAGGCAAAGATCCAGTT ^{101}TCGAGTGGTCTCTAGCATGA
^{121}AATAACCGCGAATTGGCCTT ^{141}TGTATTTCCTTCGGTGCTCC
^{161}TGCATTAGTCTCGCATGGTC8-3'

Parallel synthesis experiment
5' -^{1}TCTTCGGATCACGGTCTTTG ^{21}TGATTATTAAATCGACGCAA
^{41}CTAGGATAGAAGTGGTTAGA ^{61}TACCAGACTCCACTCTACCC
^{81}GATGTTCGTGGAAGTTGCGC^{101}TAAT-3'

5' -^{1}TCTTCGGATCACGGTCTTTG ^{21}ATGGGATCAAAGTGATACAC
^{41}AGGCCATCCTACTGTATCTA ^{61}CAATCGATACACAGGCTGGA
^{81}GGTATTCGTGGAAGTTGCGC^{101}TAAT-3'

5' -^{1}TCTTCGGATCACGGTCTTTG ^{21}GGTGCATTGACATTAGCCAA
^{41}GCGACTAGGTGCTGGCCCTA ^{61}CAATGGCTCCCGCACTGTAG

^{81}CGACTTCGTGGAAGTTGCGC^{101}TAAT-3’

5’ -^{1}TCTTCGGATCACGGTCTTTG ^{21}GGTGAATCACCTCGACACCG
^{41}CAAGCTTTGGTGCGTGAATC ^{61}ACCTATAGCACCGGCAGGGC
^{81}CATCTTCGTGGAAGTTGCGC^{101}TAAT-3’

The four sequences were converted into a single “master sequence” that defined the fluid to be delivered at each cycle of synthesis. Selective electrochemical deprotection of the growing oligonucleotide over each of the four anodes differentiates this master sequence into the four individual sequences above. In keeping with the standard established above, this master sequence is written 5’ -3’.

5’ -^{1}TCTTCGGATCACGGTCTTTG ^{21}GGTGATGATGCTGACTACTA
^{41}CTGACACTGCTGACTGACAC ^{61}GACTGATGCTGACTGATGAC
^{81}GACTGACTGATGACTACTAC ^{101}TGACTACTGACTGACTGAC
^{121}TGACGACGACTGCTGACTG ^{141}ACTGACGACTGACTTCGTG
^{161}GAAGTTGCGCTAAT8-3’

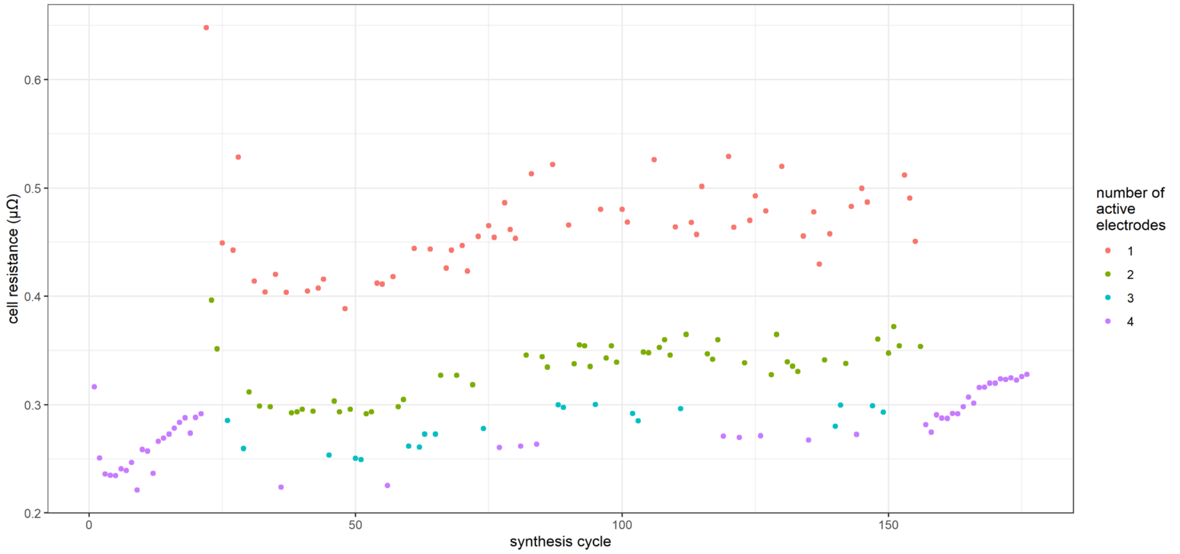

Fig. S8: **Cell resistance as a function of synthesis cycle.** The minimum observed cell resistance during a synthesis cycle was found to be correlated with the number of synthesis cycles, correcting for the number of electrodes activated during that cycle, with r = 0.33 nΩ/cycle and p = 5.6e-13.

Synthesis results: Once the synthesis protocol was complete, DNA was cleaved off the surface of the chip using 32% ammonium hydroxide and deprotected overnight at 65°C. The solution was then concentrated to dryness in a SpeedVac vacuum concentrator, followed by resuspension in 40 µL of H2O. The DNA was amplified using PCR and purified using a Qiagen QIAquick spin column or gel extracted as needed. The enriched DNA was then amplified a second time with primers containing random 25N overhangs, ligated, and sequenced using an Illumina NextSeq. Sequences were aligned using a modified Bitwise Majority Alignment algorithm (BMA) (*29*). More details regarding usage of the 25N overhangs, ligation protocol, sequencing preparation, and error analysis are described in Organick et al. [12].

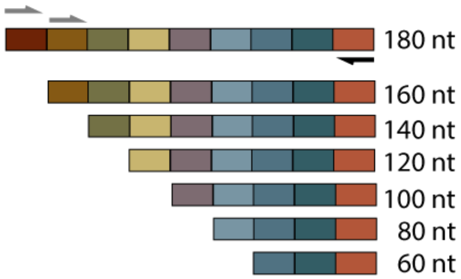

Fig. S9: **Overview of the 180 nt oligonucleotide strand with each color block representing 20 nts.** Amplification of the strand with different primer sets generate 7 different sequences. Each amplicon was then sequenced and analyzed for deletion, insertion, substitution, and read length errors.

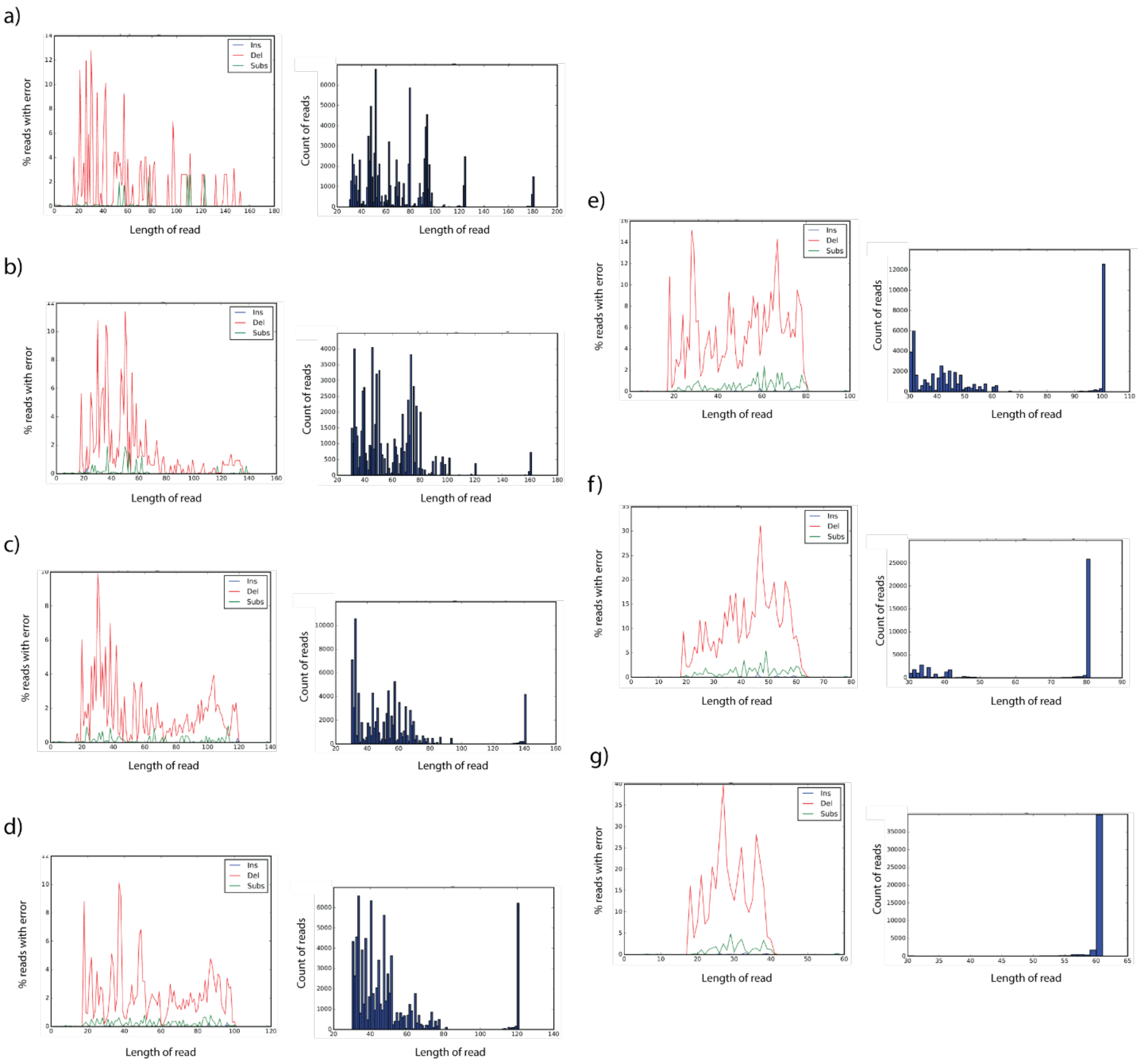

Fig. S10: **Error profiles (left) and read length analyses (right) for amplicons.** Length a) 180 nt, b) 160 nt, c) 140 nt, d) 120 nt, e) 100 nt, f) 80 nt, and g) 60 nt amplicons originate from a single 180 nt synthesis independently amplified with seven pairs of primers.

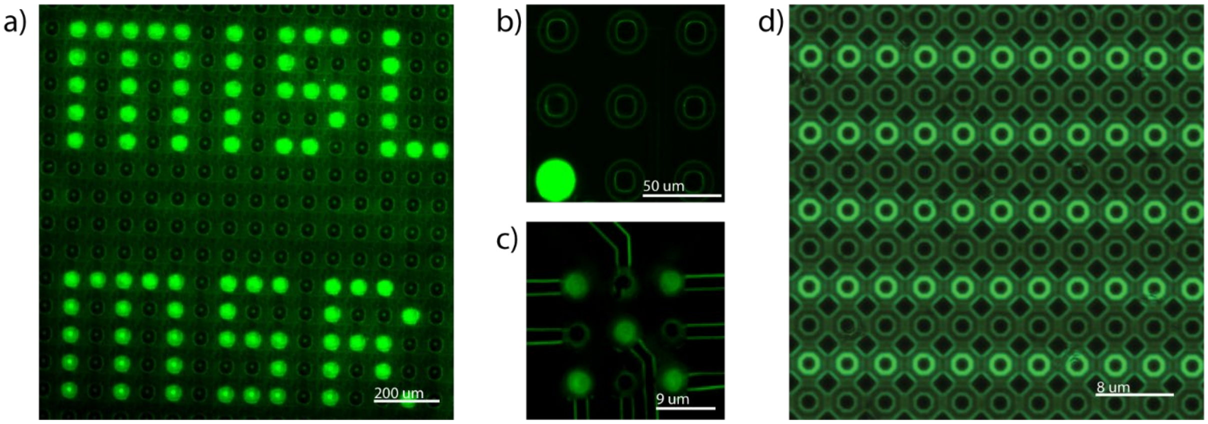

Fig. S11: **Representative images of acid-confinement experiments utilizing microelectrode arrays with larger than 650 nm diameters.** a) 50 µm electrodes in a fully addressable-array, synthesis on agarose coating. b) 25 um electrodes, synthesis on agarose coatings. c) 3 um electrodes, synthesis on agarose coating. d) 2 µm electrodes.

REFERENCES AND NOTES

1. N. Goldman, P. Bertone, S. Chen, C. Dessimoz, E. M. LeProust, B. Sipos, E. Birney, Towards practical, high-capacity, low-maintenance information storage in synthesized DNA. *Nature* **494**, 77–80 (2013).

2. Y. Erlich, D. Zielinski, DNA fountain enables a robust and efficient storage architecture. *Science* **355**, 950–954 (2017).

3. L. Ceze, J. Nivala, K. Strauss, Molecular digital data storage using DNA. *Nat. Rev. Genet.* **20**, 456–466 (2019).

4. W. D. Chen, A. X. Kohll, B. H. Nguyen, J. Koch, R. Heckel, W. J. Stark, L. Ceze, K. Strauss, R. N. Grass, Combining data longevity with high storage capacity—Layer-by-layer DNA encapsulated in magnetic nanoparticles. *Adv. Funct. Mater.* **29**, 1901672 (2019).

5. A. X. Kohll, P. L. Antkowiak, W. D. Chen, B. H. Nguyen, W. J. Stark, L. Ceze, K. Strauss, R. N. Grass, Stabilizing synthetic DNA for long-term data storage with Earth alkaline salts. *Chem. Commun.* **56**, 3613–3616 (2020).

6. L. Organick, B. H. Nguyen, R. McAmis, W. D. Chen, A. X. Kohll, S. D. Ang, R. N. Grass, L. Ceze, K. Strauss, An empirical comparison of preservation methods for synthetic DNA data storage. *Small Methods* **5**, 2001094 (2021).

7. R. K. Saiki, S. Scharf, F. Faloona, K. B. Mullis, G. T. Horn, H. A. Erlich, N. Arnheim, Enzymatic amplification of beta-globin genomic sequences and restriction site analysis for diagnosis of sickle cell anemia. *Science* **230**, 1350–1354 (1985).

8. B. H. Nguyen, J. Sinistore, J. A. Smith, P. S. Arshi, L. M. Johnson, T. Kidman, T. DiCaprio, D. Carmean, K. Strauss, Architecting Datacenters for Sustainability: Greener Data Storage using Synthetic DNA, in *2020 Electronics Goes Green 2020+ (EGG)* (2020).

9. P. M. Stanley, L. M. Strittmatter, A. M. Vickers, K. C. K. Lee, Decoding DNA data storage for investment. *Biotechnol. Adv.* **45**, 107639 (2020).

10. G. M. Church, Y. Gao, S. Kosuri, Next-generation digital information storage in DNA. *Science* **337**, 1628–1628 (2012).

11. R. N. Grass, R. Heckel, M. Puddu, D. Paunescu, W. J. Stark, Robust chemical preservation of digital information on DNA in silica with error-correcting codes. *Angew. Chem. Int. Ed.* **54**, 2552–2555 (2015).

12. L. Organick, S. D. Ang, Y.-J. Chen, R. Lopez, S. Yekhanin, K. Makarychev, M. Z. Racz, G. Kamath, P. Gopalan, B. Nguyen, C. N. Takahashi, S. Newman, H.-Y. Parker, C. Rashtchian, K. Stewart, G. Gupta, R. Carlson, J. Mulligan, D. Carmean, G. Seelig, L. Ceze, K. Strauss, Random access in large-scale DNA data storage. *Nat. Biotechnol.* **36**, 242–248 (2018).

13. I. Hirao, M. Kimoto, Unnatural base pair systems toward the expansion of the genetic alphabet in the central dogma. *Proc. Jpn Acad. Ser. B* **88**, 345–367 (2012).

14. J. Bornholt, R. Lopez, D. M. Carmean, L. Ceze, G. Seelig, K. Strauss, A DNA-based archival storage system. *SIGPLAN Not.* **51**, 637–649 (2016).

15. S. M. H. Tabatabaei Yazdi, Y. Yuan, J. Ma, H. Zhao, O. Milenkovic, A rewritable, random-access DNA-based storage system. *Sci. Rep.* **5**, 14138 (2015).

16. K. J. Tomek, K. Volkel, A. Simpson, A. G. Hass, E. W. Indermaur, J. M. Tuck, A. J. Keung, Driving the scalability of DNA-based information storage systems. *ACS Synth. Biol.* **8**, 1241–1248 (2019).

17. S. Kosuri, G. M. Church, Large-scale de novo DNA synthesis: Technologies and applications. *Nat. Methods* **11**, 499–507 (2014).

18. A. R. Pawloski, G. McGall, R. G. Kuimelis, D. Barone, A. Cuppoletti, P. Ciccolella, E. Spence, F. Afroz, P. Bury, C. Chen, C. Chen, D. Pao, M. Le, B. McGee, E. Harkins, M. Savage, S. Narasimhan, M. Goldberg, R. Rava, S. P. A. Fodor, Photolithographic synthesis of high-density DNA probe arrays: Challenges and opportunities. *J. Vac. Sci. Technol. B* **25**, 2537–2546 (2007).

19. O. D. Negrete, F. Cerrina, Step-and-scan maskless lithography for ultra large scale DNA chips. *Microelectron. Eng.* **85**, 834–837 (2008).

20. X. Li, B. Liu, B. Pei, J. Chen, D. Zhou, J. Peng, X. Zhang, W. Jia, T. Xu, Inkjet bioprinting of biomaterials. *Chem. Rev.* **120**, 10793–10833 (2020).

21. R. D. Egeland, E. M. Southern, Electrochemically directed synthesis of oligonucleotides for DNA microarray fabrication. *Nucleic Acids Res.* **33**, e125 (2005).

22. K. Maurer, J. Cooper, M. Caraballo, J. Crye, D. Suciu, A. Ghindilis, J. A. Leonetti, W. Wang, F. M. Rossi, A. G. Stöver, C. Larson, H. Gao, K. Dill, A. McShea, Electrochemically generated acid and its containment to 100 micron reaction areas for the production of DNA microarrays. *PLOS ONE* **1**, e34 (2006).

23. B. R. Takulapalli, J. Qiu, D. M. Magee, P. Kahn, A. Brunner, K. Barker, S. Means, S. Miersch, X. Bian, A. Mendoza, F. Festa, K. Syal, J. Park, J. LaBaer, P. Wiktor, High density diffusion-free nanowell arrays. *J. Proteome Res.* **11**, 4382–4391 (2012).

24. M. R. Batistuti, P. R. Bueno, M. Mulato, The importance of the assembling of DNA strands on the performance of electrochemical genosensors. *Microchem. J.* **159**, 105358 (2020).

25. W. H. Press, J. A. Hawkins, S. K. Jones, J. M. Schaub, I. J. Finkelstein, HEDGES error-correcting code for DNA storage corrects indels and allows sequence constraints. *Proc. Natl. Acad. Sci. U.S.A.* **117**, 18489–18496 (2020).

26. S. R. Srinivasavaradhan, S. Gopi, H. D. Pfister, S. Yekhanin, Trellis BMA: Coded trace reconstruction on IDS channels for DNA storage. arXiv:2107.06440 (2021).

27. P. L. Antkowiak, J. Lietard, M. Z. Darestani, M. M. Somoza, W. J. Stark, R. Heckel, R. N. Grass, Low cost DNA data storage using photolithographic synthesis and advanced information reconstruction and error correction. *Nat. Commun.* **11**, 5345 (2020).

28. S. M. Yekhanin, M. Z. Racz, Trace Reconstruction from Reads with Indeterminant Errors (2020); www.freepatentsonline.com/y2020/0057838.html.

29. T. Batu, S. Kannan, S. Khanna, A. McGregor, in *Proceedings of the Fifteenth Annual ACM-SIAM Symposium on Discrete Algorithms, SODA ‘04* (2004), pp. 910–918.

30. C. N. Takahashi, B. H. Nguyen, K. Strauss, L. Ceze, Demonstration of end-to-end automation of DNA data storage. *Sci. Rep.* **9**, 4998 (2019).

31. M. Caraballo, Oligo Pools: Design, Synthesis, and Research Applications (2018); www.genscript.com/gsfiles/techfiles/Oligo-Pools-design-synthesis-and-research-applications-slides.pdf.

32. “Massively parallel peptide synthesis on a microchip based on the µParaflo™ microfluidics technology” (Technical Bulletin MM-30–13B, 2007); www.lcsciences.com/documents/massively_parallel_peptide_synthesis_B.pdf.

33. Y. Dong, F. Sun, Z. Ping, Q. Ouyang, L. Qian, DNA storage: Research landscape and future prospects. *Natl. Sci. Rev.* **7**, 1092–1107 (2020).

34. B. H. Nguyen, D. Kesselring, E. Tesfu, K. D. Moeller, Microelectrode arrays: A general strategy for using oxidation reactions to site selectively modify electrode surfaces. *Langmuir* **30**, 2280–2286 (2014).
